# A case of adrenal cavernous hemangioma resected due to tumor growth accompanied by intratumoral hemorrhage

**DOI:** 10.1002/iju5.12760

**Published:** 2024-07-09

**Authors:** Takayuki Ueda, Masato Yanagi, Takashi Kusakabe, Takeshi Shigihara, Mikio Shibasaki, Masato Nagasawa, Tsutomu Hamasaki, Yukihiro Kondo

**Affiliations:** ^1^ Department of Urology Aidu Chuo Hospital Aizuwakamatsu‐City Fukushima Japan; ^2^ Department of Urology Nippon Medical School Hospital Bunkyo‐ku Tokyo Japan; ^3^ Department of Pathology Aidu Chuo Hospital Aizuwakamatsu‐City Fukushima Japan; ^4^ Department of Radiology Aidu Chuo Hospital Aizuwakamatsu‐City Fukushima Japan; ^5^ Department of Urology Nippon Medical School Musashikosugi Hospital Kawasaki‐City Kanagawa Japan

**Keywords:** adrenal tumor, adrenalectomy, cavernous hemangioma, intratumoral hemorrhage, laparoscopic

## Abstract

**Introduction:**

We describe a case of an adrenal cavernous hemangioma that was surgically resected because of tumor growth and intratumoral hemorrhage.

**Case presentation:**

A 73‐year‐old woman presented with an enlarged adrenal tumor and intratumoral hemorrhage during the follow‐up of an incidental adrenal tumor. A computed tomography showed that the left adrenal tumor had grown from 23 to 44 mm over 1 year. Blood tests revealed a normal metabolic profile. Paragangliomas and metastatic tumors were suspected on imaging. Laparoscopic adrenalectomy was performed to prevent tumor rupture due to further bleeding. No adhesions or bleeding were observed around the tumor during surgery. Pathological diagnosis was adrenal cavernous hemangioma.

**Conclusion:**

Adrenal cavernous hemangioma is difficult to distinguish preoperatively from other adrenal tumors, including malignant tumors. The intraoperative findings of this case suggest that laparoscopic adrenalectomy is a safe treatment option for relatively small adrenal cavernous hemangioma.

**Abbreviations & Acronyms**
ACH: adrenal cavernous hemangiomas
CT: computed tomography
MRI: magnetic resonance imaging

**Keynote message**

We describe a case of an adrenal cavernous hemangioma adrenal cavernous hemangioma that was surgically resected because of tumor growth and intertumoral hemorrhage. The intraoperative findings of this case suggest that laparoscopic adrenalectomy is a safe treatment option for relatively small adrenal cavernous hemangioma.

## Introduction

The detection rate of adrenal tumors has increased with the widespread use of imaging techniques such as CT, MRI, and ultrasonography. As a result, various types of adrenal tumors, including ACH have been discovered. ACH is a collection of vascular malformations arising from the endothelial layer of blood vessels and was first described by Johnson *et al*. in 1955.^1^ Since then, only 76 cases have been reported to date.^2^, ^3^, ^4^, ^5^, ^6^, ^7^ Most ACHs are nonfunctional tumors, but rarely they are functional. In addition, ACHs are rarely associated with retroperitoneal hemorrhage, which can be life‐threatening.^5^, ^6^

In this report, we describe a case of ACH that was surgically resected due to tumor growth and intratumoral hemorrhage.

## Case presentation

**Fig. 1:**
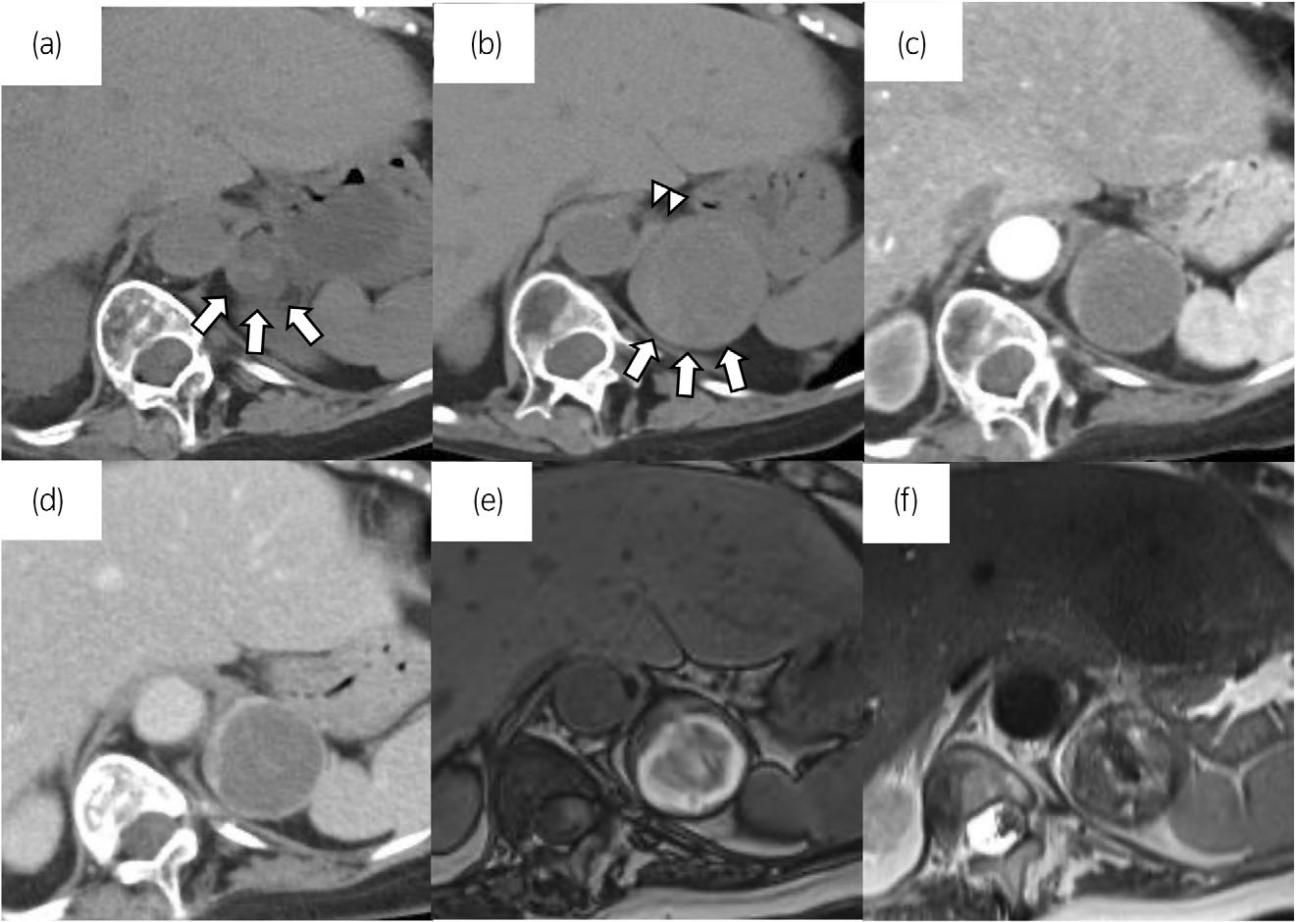
Findings of CT and MRI. (a) Plain CT 1 year before surgery. A CT scan showed a 23‐mm left adrenal tumor that was diagnosed as an adrenal cortical adenoma. White arrows indicate the adrenal tumor. (b) Plain CT. White arrowheads indicate the left adrenal gland. White arrows indicate the adrenal tumor. Calcification of the tumor was not observed. (c) Arterial phase on contrast‐enhanced CT. (d) Portal phase of contrast‐enhanced CT. The tumor margins are clearly contrasted, and the inside of the tumor is lightly contrasted. (e, f) On MRI, an area inside the tumor with high intensity on T1‐weighted images (e) and low intensity on T2‐weighted images (f) is observed, and intratumoral hemorrhage is suspected.

**Fig. 2:**
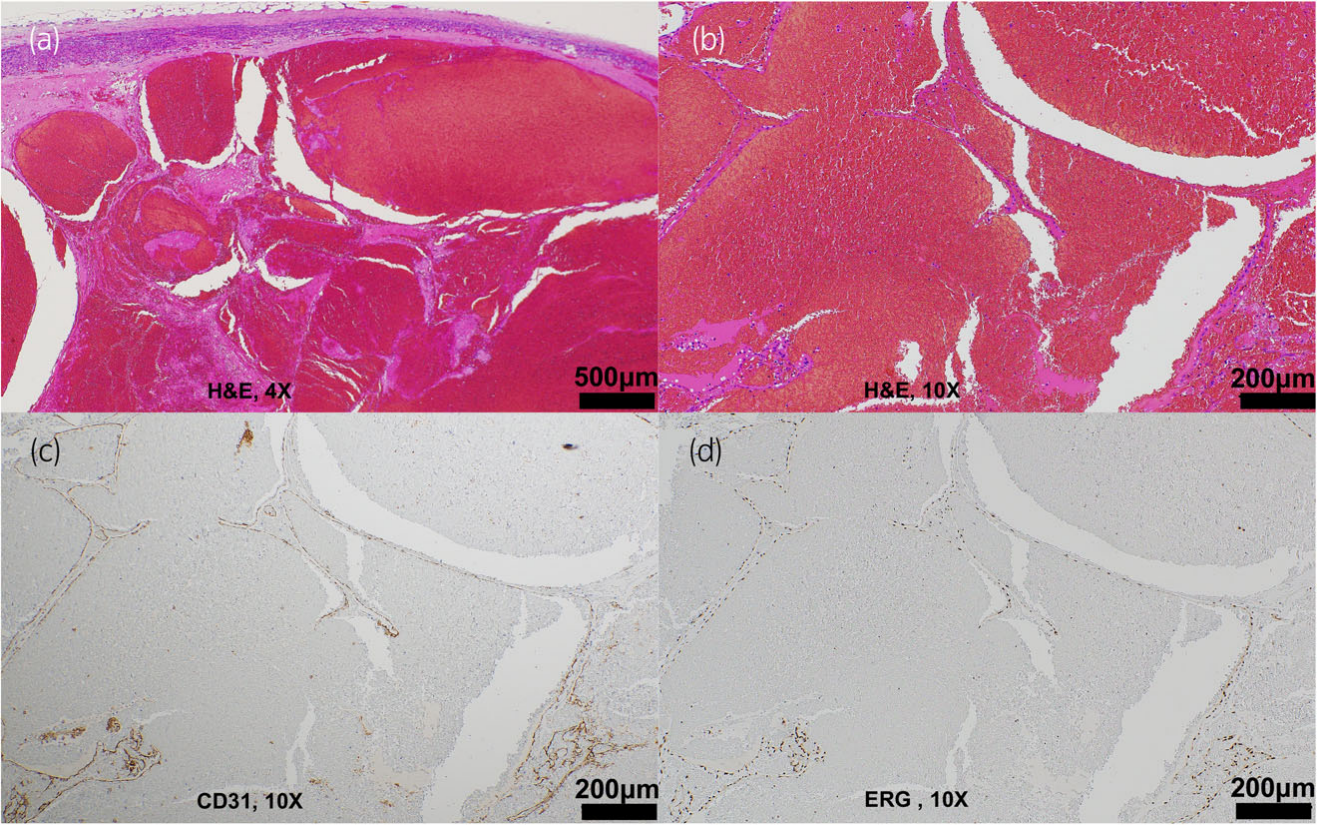
Pathological findings of the tumor. (a, b) Thin‐walled cavernous blood vessels are observed at the margin of the tumor. (c) Immunostaining confirmed CD31 positivity in the cytoplasm of the vascular endothelium. (d) Immunostaining confirmed ERG positivity in the nuclei of the vascular endothelium.

A 73‐year‐old woman presented with an enlarged tumor and intratumor hemorrhage during the follow‐up of an incidental adrenal tumor. One year prior, a CT scan showed a 23‐mm left adrenal tumor that was diagnosed as an adrenal cortical adenoma (Fig. 1a). Follow‐up CT revealed that the left adrenal tumor had grown to 44 mm. Serum levels of catecholamines, renin, aldosterone, adrenocorticotropic hormone, and cortisol were within normal ranges. Contrast‐enhanced CT revealed that the tumor had peripheral patchy enhancement, and its internal structure was lightly contrasted (Fig. 1b–d). On MRI, an area inside the tumor with high intensity on T1‐weighted images and low intensity on T2‐weighted images was observed, and an intratumor hemorrhage was suspected (Fig. 1e, f). In addition, 123I‐metaiodobenzylguanidine scan was negative. Paraganglioma and metastatic tumors were suspected as differential diagnoses based on the contrast‐enhanced CT and MRI findings. The patient had a history of cerebral infarction due to atrial fibrillation and was taking edoxaban tosilate hydrate and aspirin. The patient had no history of abdominal trauma. Although it was an asymptomatic and nonfunctional adrenal tumor, it grew with internal bleeding inside the tumor, and malignancy could not be ruled out. The patient required permanent anticoagulation therapy. Because malignancy could not be ruled out and to prevent tumor rupture due to further bleeding, laparoscopic adrenalectomy was performed to prevent tumor rupture due to further bleeding. The operation time was 126 min, and blood loss was 2 mL. No post‐operative complications were observed. The patient was discharged on postoperative day 9. The tumor was a 50 × 40 × 35‐mm mass with clear borders, and most of the inside of the tumor was a clot. Pathologically, thin‐walled cavernous blood vessels were observed at the tumor margins (Fig. 2a, b). Immunostaining confirmed that vascular endothelial markers (CD31, ERG) were positive on the inner surface of the cavernous blood vessels (Fig. 2c, d). Based on these findings, the patient was diagnosed with ACH.

## Discussion

ACH is a collection of vascular malformations arising from the endothelial layer of blood vessels. It is considered that ACH do not regress spontaneously and continue to enlarge over time as a result of proliferation of hemangioma and repeated intratumoral hemorrhage. According to past reports,^2^, ^3^, ^4^, ^5^, ^6^, ^7^ the median age of patients with ACH was 63 years, with a male‐to‐female ratio of approximately 3:2 and slightly more females affected (Table 1). ACHs were found incidentally in 59.2% of the cases. The symptoms often include pain and discomfort in the lower back and abdomen. Most are nonfunctioning tumors; however, rare cases of endocrine dysfunction, such as aldosteronism, have been reported.

**Table 1:** Summary of the ACH reported between 1955 and 2023

Characteristics	Data (*n* = 76)
Median age; year (range)	63 (19–84)
Sex	
Male	33 (43.4%)
Female	43 (56.6%)
Laterality	
Right	37 (48.7%)
Left	39 (51.3%)
Median tumor size; mm (range)	90 (20–270)
Clinical presentation	
Asymptomatic	45 (59.2%)
Symptomatic	31 (40.8%)
Frank pain or discomfort	8 (10.5%)
Abdominal symptoms	10 (13.2%)
Retroperitoneal hemorrhage	3 (3.9%)
Others	10 (13.2%)
Metabolic profile	
Normal	70 (92.1%)
Abnormal	6 (7.9%)
Hyperaldosteronism	3 (4.0%)
Subclinical Cushing's syndrome	3 (4.0%)
Speckled calcification	
Present	34 (44.7%)
Absent	32 (42.1%)
Unknown	10 (13.2%)
Management/non‐surgical management	
Non‐surgical management	1 (1.3%)
Surgical management	75 (98.7%)
Open	54 (71.1%)
Laparoscopic	19 (25.0%)
Unknown	2 (2.6%)

About half the cases with ACH show a heterogeneous internal structure of mass on plain CT.^7^ In addition, about half the cases with ACH show peripheral patchy enhancement or a centripetal pattern of enhancement of the mass on contrast‐enhanced CT.^7^ Moreover, calcification is seen in about half the cases with ACH on CT.^7^ In the present case, the adrenal tumor showed a heterogeneous internal structure on plain CT and peripheral patchy enhancement on contrast‐enhanced CT but did not have calcification. On MRI, ACH tends to show low intensity on T1‐weighted images.^8^, ^9^ In the present case, the tumor showed high intensity on T1‐weighted images because of the hemorrhage. CT and MRI findings of ACH overlap with those of various adrenal diseases, such as cancer, cysts, tuberculosis, neuroblastoma, and metastatic tumors.^7^, ^8^, ^9^ Therefore, it is not easy to diagnose ACH based on preoperative CT and MRI findings.

Almost all cases, including the present case, were diagnosed via surgical resection. As ACH is rare, optimal treatment guidelines have not yet been established. However, when the tumor is large (>6 cm) and malignancy cannot be ruled out, when it is symptomatic or when it is functional, surgical resection is indicated. Although rare, surgery is indicated when retroperitoneal hemorrhage is present. In this case, permanent anticoagulant administration was necessary. Even if spontaneous hemostasis could be achieved in this case, retroperitoneal hemorrhage due to tumor rupture could be fatal in the future; therefore, we resected the tumor. However, when the tumor is small, asymptomatic, and nonfunctioning, active surveillance may be considered.^2^

Before 2000, all adrenalectomies for ACH were performed by laparotomy, but since 2010, laparoscopic surgery has become more common owing to the development of medical technology.^2^ In the present case, the laparoscopic procedure was not hemorrhagic, and the procedure did not seem to be more difficult than for other adrenal tumors. Noh *et al*. maintained that a laparoscopic approach is feasible and safe to remove ACH because ACH tend to form a rigid fibrotic capsule resulting in the low risk of bleeding due to surgical manipulation.^7^

In conclusion, ACH is difficult to distinguish preoperatively from other adrenal tumors, including malignancies. ACH might require surgical resection because of the risks, including the possibility of malignancy, symptoms, retroperitoneal hemorrhage, and intertumoral hemorrhage. Furthermore, laparoscopic adrenalectomy may be considered a safe treatment option for ACH.

## Author contributions

Takayuki Ueda: Conceptualization; data curation; writing – original draft. Masato Yanagi: Conceptualization; data curation; writing – original draft. Takashi Kusakabe: Data curation. Takeshi Shigihara: Data curation. Mikio Shibasaki: Data curation. Masato Nagasawa: Data curation. Tsutomu Hamasaki: Data curation; writing – original draft. Yukihiro Kondo: Writing – original draft; writing – review and editing.

## Conflict of interest

The authors declare no conflict of interest.

## Approval of the research protocol by an Institutional Reviewer Board

Not applicable.

## Informed consent

Written informed consent was obtained from the patient.

## Registry and the Registration No. of the study/trial

Not applicable.
